# How shared suffering bonded Britons witnessing the Queen’s funeral

**DOI:** 10.1038/s41598-024-66537-5

**Published:** 2024-07-18

**Authors:** Claire White, Danielle Morales, Dimitris Xygalatas, Mathilde Hernu, Anna Mathiassen, Andrew Ainsworth, Meara Geraty, Nisa Bayindir, Brooke Robinson, Harvey Whitehouse

**Affiliations:** 1 Religious Studies Department, California State University Northridge, 230 Santa Susana Hall, 18111 Nordhoff Street, Northridge, CA 91330-8316 USA; 2 Department of Sociology, California State University, Northridge, USA; 3 Department of Anthropology, University of Connecticut, Storrs, USA; 4 Department of Psychological Sciences, University of Connecticut, Storrs, USA; 5 Institute of Cognition and Culture, Queen’s University, Belfast, UK; 6 Department of Psychology, Aarhus University, Aarhus, Denmark; 7 Department of Psychology, California State University, Northridge, USA; 8 Department of English, Queen’s University, Belfast, UK; 9 School of Anthropology & Museum Ethnography, Oxford University, Oxford, UK

**Keywords:** Social bonding, Identity fusion, Funeral, Prosociality, Ritual, Monarchy, British public, Psychology, Human behaviour

## Abstract

Previous research suggests that sharing emotionally intense experiences with others, for example by undergoing dysphoric collective rituals together, can lead to “identity fusion,” a visceral feeling of oneness that predicts group cohesion and self-sacrifice for the group. In this pre-registered research, we provide the first quantitative investigation of identity fusion following participation in a national funeral, surveying 1632 members of the British public. As predicted, individuals reporting intense sadness during Queen Elizabeth II’s funeral exhibited higher levels of identity fusion and pro-group commitment, as evidenced by generosity pledges to a British Monarchist charity. Also consistent with our hypotheses, feelings of unity in grief and emotional sharedness during the event mediated the relationship between sadness intensity and pro-group commitment. These findings shed light on importance of collective rituals in fostering group cohesion, cooperation, and the dynamics of shared emotional experiences within communities.

## Introduction

On 19th September 2022, around a million people lined the streets of London to witness the funeral procession of Britain's longest-serving Monarch, Queen Elizabeth II^1^. 37.5 million people in the UK—and tens of millions more globally—watched the event live on screens^2,3^. The historic ritual procession was characterized by closely choreographed coordination and pageantry steeped in symbols of Britishness. Attendees noted the somber atmosphere, stark silence, and sense of shared loss among the vast crowds^4^. Some wept, others clung to each other, and crowds spontaneously applauded as a mark of gratitude as the flag-draped oak coffin passed by^5^. British news outlets reported a nation united in grief^6^.

These reports align with contemporary research on the social psychology of group commitment. Accumulating evidence reveals that sharing personally transformative experiences with others, especially ones that evince powerful emotions and lasting memories, leads to a form of very strong social bonding in which personal and group identities become fused together^7–9^. This form of group alignment is known as ‘identity fusion’^10^ and is associated with extreme forms of pro-group commitment, ranging from acts of charity^11^ to willingness to sacrifice one’s life^12^.

**Figure 1:**
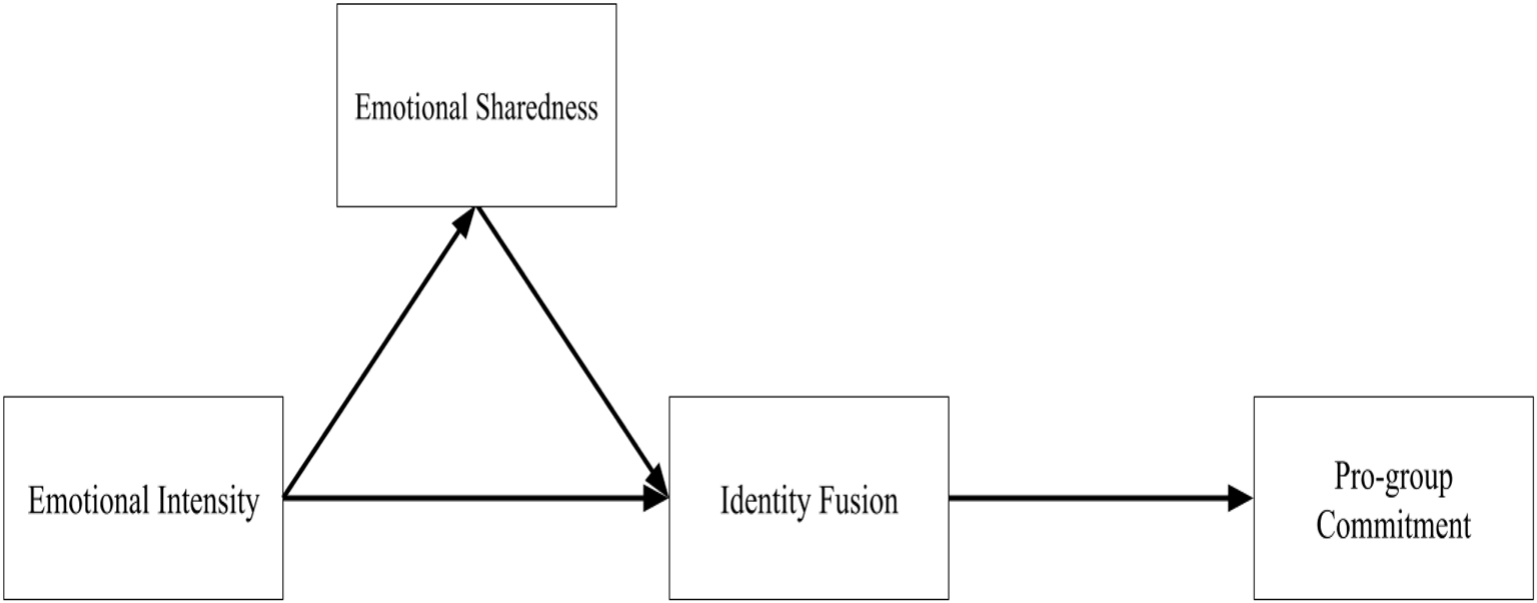
Theoretical model of the shared-experiences pathway to fusion and its behavioral outcomes (based on^10^ Whitehouse, 2018).

We introduce a novel contribution to the literature on social bonding by conducting the first investigation into the effects of participating in a collective mourning ritual on identity fusion. While previous research has provided evidence for the shared-experiences-pathway to identity fusion (see Fig. 1) across various groups facing potent dysphoric ordeals, such as Bostonians following the 2013 Marathon Bombings^13^ and Libyan insurgents during the 2011 conflict^14^, our study represents the inaugural exploration into the impact of funerary rituals on fusion dynamics. Despite a rich qualitative tradition in the social sciences indicating that funerary rites foster and strengthen group cohesion^15–19^, quantitative investigations into the effects of funerary processions on group psychology have been scarce. As a result, our knowledge of this universal human behavior with potentially far-reaching implications for social organization remains limited. Our study aims to address this gap by employing psychometric measures to assess the impact of funerary rituals on identity fusion.

## The present study

We began our enquiry with four basic questions: (1) Did the reported intensity of sadness during the Queen’s funeral facilitate psychological bonding among fellow attendees? (2) What was the relationship between the perceived intensity of shared suffering and the extent to which attendees felt fused with others? (3) was strength of identity fusion amongst attendees associated with charitable behavior towards British Monarchists? And (4) did fusion towards fellow attendees extend to all members of the British public who mourned the death of the Queen?

Based on the ‘shared experiences pathway to fusion’ theory, we predicted (1) that reported levels of the intensity of sadness during the live state funeral would predict identity fusion with co-attendees, and (2) the relationship between emotional intensity and fusion would be mediated by perceived sense of sharedness of emotions and the grief experience. To capture shared experience among participants in the funeral, we used two novel measures based on the funeral context (unity in grief, shared emotional pain) to ascertain whether and how shared sadness during the ceremony impacted fusion.

Finally, we expected that (3) fusion levels with co-attendees would predict engagement in self-sacrificing, pro-group commitment (charitable pledges) towards British Monarchists, and that (4) these findings (1–3) would also be evidenced towards members of the British public.

Moreover, based on previous research suggesting that the effects of shared experiences on fusion are stronger in relational groups than categorical groups (e.g.,^14^), we hypothesized that (5) fusion levels would be highest for co-attendees (i.e., those who viewed or attended the ceremony together, whether on screen or in person on the streets as the procession passed) compared with members of the British public as a more abstract and generic group category of people.

## Methods

All studies were conducted in accordance with and approved by California State University Northridge IRB, Committee for Protection of Human Subjects (CPHS), IRB-FY23-76. The study was performed in compliance with the ethical guidelines provided by CPHS at California State University, Northridge. Digital consent was obtained from all participants. This research is preregistered: https://aspredicted.org/x85qn.pdf.

### Participants

A total of 1632 participants were included in the study. They were: British residents of 18 years of age or older who met inclusion requirements (submitted once, took more than five minutes to complete the survey, completed the survey within the specified time frame, and passed Qualtrics’ fraud detection measures), watched the funeral live (physically or via live stream), and reported remembering their experience of the Queen’s funerary processions. After these criteria were applied, responses from 1727 participants were excluded from the 3359 surveys submitted (51% of participants excluded were bots identified by fraud detection in Qualtrics).

Recruitment took place during the first two weeks following the state funeral. For all studies, participants were recruited in person by the study team around public spaces in Central London (e.g., markets, parks, the queue outside Westminster Abbey) and online via UK Facebook group pages likely to have members who viewed the funeral ceremony (e.g., containing the words “Queen Elizabeth II”).

Participants identified as 61.6% male and 38.3% female (0.1% preferred not to say) with an average age of 32.9 (*SD* = 8.84). Most participants (70.7%) identified as White or Euro-American (10.6% Black, 7% Asian, 6.7% Hispanic or Latino, 4.3% Middle Eastern, 0.8% other/prefer not to say). The majority of respondents (69.5%) resided in England, followed by Ireland (14.3%), Scotland (9.1%), Wales (5.5%), and Northern Ireland (1.5%). Two-thirds of participants (66.7%) viewed the funeral virtually via live-streaming and a third (33.3%) attended physically.

### Design

#### Materials and procedure

Participants completed an online survey hosted on Qualtrics by scanning a QR code or following a link on flyers. We did not collect identifying information such as names or addresses. The first 300 participants who met the inclusion criteria were emailed a £15 Amazon gift card. The survey consisted of thirty-two questions and took participants eleven minutes, on average, to complete.

##### Emotional intensity

Participants rated the emotional intensity of their sadness during the funeral ceremony on a scale of 0 (not intense at all) to 4 (extremely intense).

##### Sharedness

The sharedness of the experience was assessed by two measures: unity in grief and emotional sharedness (see below).

##### Unity in grief

Unity in grief was measured with a single item “during the ceremony, I felt that people were united in grief with me” and response options ranging from 0 (strongly disagree) to 4 (strongly agree).

##### Emotional sharedness

Emotional sharedness with each group was measured with a single question “do you think your emotional pain has been lesser, greater, or the same as [for the coatendees target group: the people who attended the funeral with you/for the British public target group: typical person who mourned the loss of the Queen]?” participants who viewed the ceremony alone were not asked the question about sharedness with co-attendees. This item was (rated on a scale) on a scale from 1 (much less painful), with an intermediate (shared) score of 3 (about the same) to 5 (much more painful). The original coding measured relative pain, with higher scores indicating more pain and lower scores indicating less pain. These variables were therefore re-coded for analysis so that the numerical scores directly corresponded to the level of sharedness on a 3-point scale with 0 representing the least amount of emotional sharedness (reports of much more or much less painful than others) and 2 signifying the greatest perceived similarity (about the same as others).

##### Identity fusion

**Figure 2:**
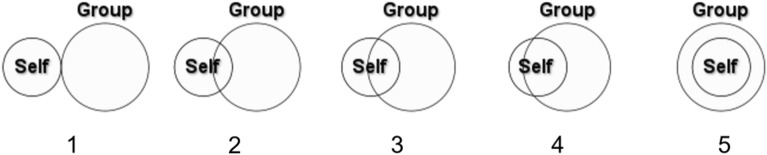
Pictorial measure of fusion.

Identity fusion was measured using the pictorial measure of fusion^20^ as displayed in Fig. 2, with two target groups: (a) co-attendees and (b) the British public. The self is depicted as a small circle and the group as a larger circle. The two circles overlap to varying degrees in five options, and participants select which image best characterizes their relationship with the group. The images correspond to different levels of fusion, with the last image (5), where the small circle (personal self) is entirely enclosed by the big circle (group) representing the strongest degree of fusion.

##### Progroup commitment

We measured progroup commitment as the number of British pounds that participants were willing to donate from their participation gift card, indicated on a sliding scale ranging from £0–£15 in £1 increments. Participants read, “if you receive £15 for participating in this study, then how much would you be willing to donate to a new non-profit charity…The Preservation of the British Monarchy?”. The charity’s mission was described as “help[ing] preserve the British Monarchy by promoting awareness of how important they are to the identity and legacy of the United Kingdom.” Participants were unaware that this item was hypothetical (i.e., the charity was fictional and the amount they pledged was not deducted from their total compensation).

## Results

Mean emotional intensity of sadness levels among participants were high (*M* = 2.99, *SD* = 1.08), as were scores on perceived unity in grief during the funeral (*M* = 2.97, *SD* = 0.92). Over a third of participants reported the highest emotional sharedness during the ceremony with co-attendees and the British public (35.3% and 36.7%, respectively). As expected, fusion levels towards those present during the ceremony and towards the British public were moderately correlated (*r*(1106) = 0.483, *p* < 0.001), with fusion levels with co-attendees rated significantly higher than the British public (co-attendees, *M* = 3.80, *SD* = 1.02; British public,* M* = 3.57, SD = 1.05), dependent samples *t*(1105) = 3.77, *p* < 0.001. On average, participants were willing to donate £7.36 (SD = 4.64) to support the British Monarchy.

When considering the mode of witnessing the live funeral ceremony (physical or virtual), the difference in levels of sharedness was insignificant for co-attendees [*t*(1093) = − 1.60, *p* = 0.11, *d* = − 0.10] and small for the British Public [*t*(1615) = − 2.34, *p* = 0.02, *d* = − 0.12]. The intensity of sadness was significantly different between groups, with those who attended in person reporting higher levels of sadness than those who viewed online, *t*(1604) = 2.20, *p* = 0.03 (*d* = 0.12). However, due to the low effect sizes, mode of attendance was collapsed in all subsequent analyses.

As per the preregistration, we conducted separate regression models for each of the two target identity groups (co-attendees and the British public).

### Co-attendees

#### Emotional sharedness as a mediator

**Figure 3:**
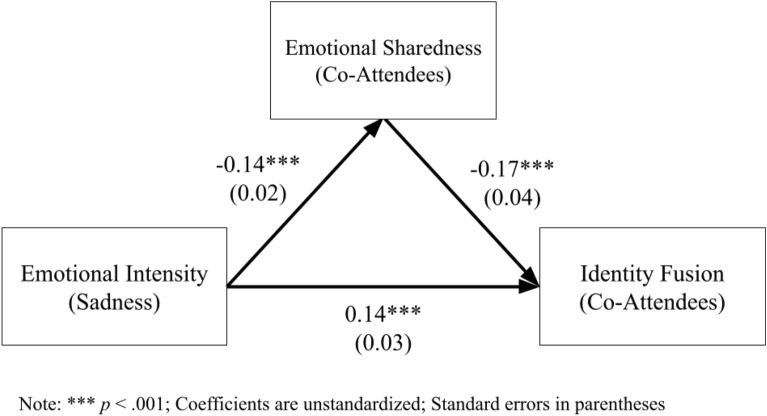
Mediation model—emotional sharedness for co-attendees.

A mediation model was created to predict identity fusion from emotional intensity via emotional sharedness. As Fig. 3 illustrates, emotional intensity significantly predicts identity fusion both directly and indirectly; the indirect effect was significant as tested by the Sobel test^21^, *z*_*sobel*_ = 3.47, *p* < 0.001.

#### Unity as a mediator

**Figure 4:**
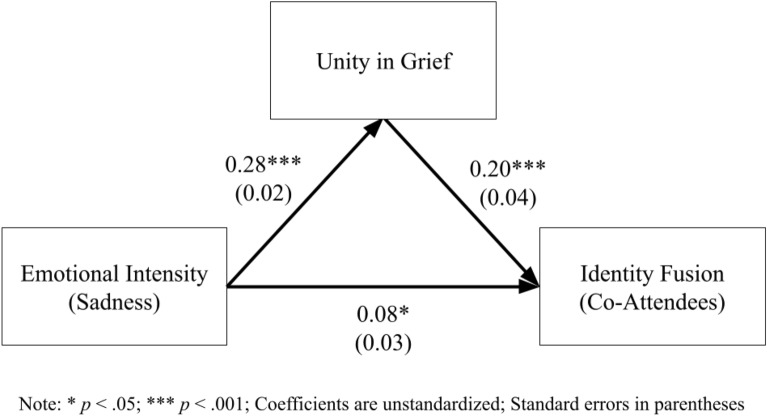
Mediation model—unity in grief for co-attendees.

A mediation model was created to predict identity fusion from emotional intensity via unity in grief. As Fig. 4 illustrates, emotional intensity significantly predicts identity fusion both directly and indirectly; the indirect effect was significant: *z*_*sobel*_ = 4.71, *p* < 0.001.

#### Fusion predicts charitable behavior

Identity fusion with co-attendees predicted charitable commitment, *b* = 0.44, *t*(1031) = 3.04, *p* = 0.002,* r*^*2*^ = 0.01. Thus, for every one unit increase in fusion, we can expect charitable donations to increase by £0.44.

### British public

#### Emotional sharedness as a mediator

**Figure 5:**
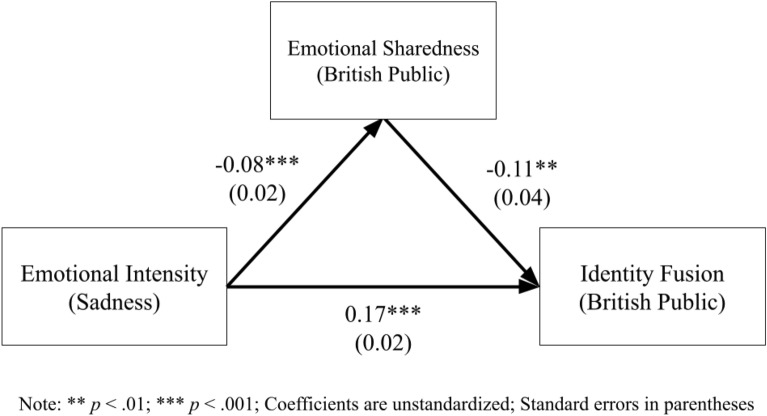
Mediation model—emotional sharedness for British public.

A mediation model was created to predict identity fusion from emotional intensity via emotional sharedness. As Fig. 5 illustrates, emotional intensity significantly predicts identity fusion both directly and indirectly; the indirect effect was significant: *z*_*sobel*_ = 2.56, *p* < 0.05.

#### Unity as a mediator

**Figure 6:**
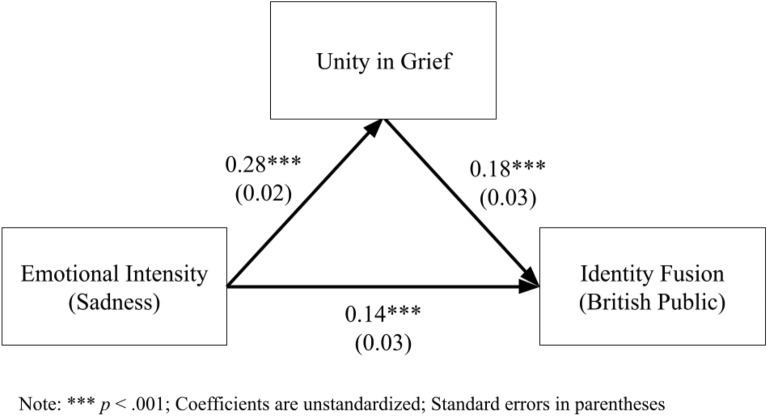
Mediation model—unity in grief for British public.

A mediation model was created to predict identity fusion from emotional intensity via unity in grief. As Fig. 6 illustrates, emotional intensity significantly predicts identity fusion both directly and indirectly; the indirect effect was significant: *z*_*sobel*_ = 5.51, *p* < 0.001.

#### Fusion predicts charitable behavior

Identity fusion with the British Public predicted charitable behavior, *b* = 1.15, *t*(1517) = 10.58, *p* < 0.001, *r*^*2*^ = 0.07. For every one unit increase in fusion, we can expect charitable donations to increase by £1.15. Unsurprisingly, the model measuring fusion levels with the British public was a stronger predictor of charity donations to preserve the legacy of the British Monarchy (i.e., essentially, donations to the British public) than fusion levels with co-attendees, who were more likely to include friends and family members.

## Discussion

As predicted in this preregistered research, among those witnessing the live funeral of Queen Elizabeth II, powerful feelings of sadness and shared emotional suffering with co-attendees and fellow Britons were associated with high levels of identity fusion with these groups. We also found evidence of differences based on the mode of witnessing the event, with perceived intensity of sadness significantly higher for those who attended the funeral in person as compared to those who viewed online. This finding is in accordance with prior research showing that physiological arousal is significantly higher and more aligned between group members during physical compared to virtual emotional events, such as devoted basketball fans watching their team play in the stadium rather than on television^22,23^.

Furthermore, attendees who were highly fused with co-attendees and members of the British public evidenced high levels of pro-group commitment: They pledged to donate higher portions of their compensation to preserve the British Monarchy’s legacy. As such, this study provides further support for the shared-experiences-pathway to fusion theory. Numerous studies have shown that shared suffering (e.g.,^7,11^) including shared feelings of bereavement (e.g.,^24^) can lead to fusion, but this is the first study to show that funerary rituals can provide a powerful institutional setting for fusion to be generated.

This supports the widely held view among social scientists that funerals are occasions for social bonding (see^25^ for an overview) and sheds light on the psychological processes involved in ways that can be specified more precisely and measured statistically for the first time.

Here, we provide the first quantifiable investigation of extreme social bonding in the aftermath of a national funeral. Moving beyond the confines of the lab to run studies in real-life settings with members of the general population involves trade-offs between control and ecological validity, particularly when researching topics of a sensitive nature involving strong emotions. Thus, the cross-sectional nature of our correlational study precludes strong causal inference. We cannot rule out an alternative interpretation of our data, i.e. that Britons who were already highly fused were more likely to feel intense sadness during the Queen’s funeral and to donate more of their funds to a British charity. However, previous research suggests that for some people the mere act of thinking about shared experiences of death and bereavement can prime feelings of fusion (e.g.,^24^), suggesting that participation in highly orchestrated outpourings of collective grief, such as national funerals, is the cause rather than consequence of fusion with other mourners. Future experimental studies can complement contextually based studies by providing further insight into the unique contribution of grief rituals (for comparable social losses) on the predicted effects of identity fusion and prosocial commitment.

Moreover, as we collected data up to two weeks after the funeral, we were not able to fully establish whether fusion with the groups and pro-group commitments were triggered solely by the emotional intensity elicited at the time of the queen's funeral or by the queen's passing more broadly. We expect that the emotional intensity of the two (i.e., reactions to the news of the queen’s death and viewing the funeral) are highly correlated but note that the absence of a pre-event assessment makes this an unanswered empirical question. Longitudinal studies are required to address these possibilities.

Additionally, we were unable to draw robust conclusions from our data about differences between fusion levels based on the mode of attendance or the group target. The naturalistic context of this study renders it impossible to isolate and pinpoint the unique contribution of these variables because they differ in more than one way. For example, those who physically attended the ceremony may have viewed digital footage later, and co-attendees were likely to entail family and friends who accompanied them, as well as members of the British public who formed crowds. Due to the practical and consequential implications of generating identity fusion en masse via digital media in the twenty-first century, further investigation into these questions is urgently needed. Experimental studies would be especially fruitful in discerning the effect of the mode of participation on social bonding.

Finally, we documented evidence of social bonding effects up to 2 weeks following the event. Previous research has demonstrated the long-term impacts of shared dysphoria on identity fusion (e.g.,^26^). Whether the effects of participating in the Queen’s funeral endure over similarly extended periods was beyond this study's scope but is worthy of future investigation in a longitudinal design. Likewise, future research on funerary rituals may also consider the extent of reflection after the ceremony as another key variable in the hypothesized psychological pathway to identity fusion^7,9^.

Overall, this research contributes to accumulating evidence that sharing emotionally intense dysphoric experiences with others, including attending sacred rituals [^27,28^], leads to strong forms of social bonding. Our research adds to a growing body of evidence in support of the ‘shared experiences pathway to fusion’ theory^29^, building on studies focusing on the effects of collective dysphoric experiences, for example among football fans^30^, martial arts practitioners^31^ and university fraternities^24^.

Although the weight of evidence that shared dysphoric experiences lead to fusion is strong^12^, some studies of large-scale political rituals such as presidential inaugurations (e.g.^32^) and events like referendum outcomes^33^ have produced mixed results. For example, Democrats in America observing the inauguration of Donald Trump and remain supporters witnessing the outcome of the Brexit referendum in the UK reported feelings of shared suffering but not fusion. One plausible explanation is that these collective events were not designed to address the psychological needs of those populations and indicated significant societal divisions – indeed Trump’s inauguration and the Brexit referendum may have been perceived by American Democrats and British ‘Remainers’ respectively as alienating events celebrating the victories of reviled outgroups. By contrast, the queen’s funeral was designed as a collective dysphoric ritual for all who witnessed it, emphasizing shared purpose as well as shared experience.

In summary, this study advances our scientific knowledge of group cohesion by elucidating the psychological pathways through which collective mourning rituals can impact social bonding. Our findings demonstrate that emotional intensity and shared grief are potent mediators in the relationship between ritual participation and identity fusion. These findings have important implications for our understanding of the societal impact of national rituals, particularly their role in reinforcing collective identities, enhancing social cohesion, and motivating altruistic tendencies and collective action. In this case, the funeral of Queen Elizabeth II served as a focal point for national identity and unity. Such events can help bridge existing divisions in society and strengthen feelings of national solidarity. Policymakers and community leaders can use these insights to leverage collective rituals to foster more effective forms of cooperation, which can be particularly valuable during periods of national mourning.

## Data Availability

Data, transcripts, and a transparent changes document are freely available online at: https://www.clairejwhite.com/s-projects-basic.
